# MODERATING EFFECT OF DEPRESSION ON THE ASSOCIATION OF COGNITIVE IMPAIRMENT WITH EVERYDAY FUNCTION IN OLDER ADULTS

**DOI:** 10.1093/geroni/igae098.3317

**Published:** 2024-12-31

**Authors:** Thomas McIntyre, Jacqueline Becker, Katherine Hackett, Lili Chan, Fernando Carnavali, Juan Wisnivesky, Laura Curtis, Alex Federman

**Affiliations:** Ichan School of Medicine at Mount Sinai, New York, New York, United States; Ichan School of Medicine at Mount Sinai, New York, New York, United States; Icahn School of Medicine at Mount Sinai, New York, New York, United States; Ichan School of Medicine at Mount Sinai, New York, New York, United States; Ichan School of Medicine at Mount Sinai, New York, New York, United States; Ichan School of Medicine at Mount Sinai, New York, New York, United States; Northwestern University Feinberg School of Medicine, Chicago, Illinois, United States; Icahn School of Medicine at Mount Sinai, New York, New York, United States

## Abstract

Cognitive impairment (CI) and depression often co-occur, and each is known to impair older adults’ ability to perform activities of daily living. We sought to determine whether co-morbid depression moderates the association of CI with impairments in instrumental activities of daily living (IADL). We recruited English speaking adults ages ≥55 years without preexisting dementia or CI from primary care practices in New York City and Chicago (n=1,045). IADLs were assessed using Lawton’s 8-item scale and impairment was defined as ≥1 activity requiring assistance. Cognitive function was measured using Montreal Cognitive Assessments (MoCA); moderate-severe CI was defined as MoCA score ≥1.5 standard deviations below the age and education adjusted z-score. Depression was defined as a score ≥10 using Patient Health Questionnaires (PHQ)-8. We fit linear regression models estimating the association of mild CI, moderate-severe CI, and depression with counts of IADL impairments, adjusting for age and sex. We then added an interaction term for CI and depression. The mean age was 67 (8.0) years, 54% were female, 35% Black and 25% Hispanic. Median IADL impairments were 0 (IQR 0-7); 16% had mild CI, 5% moderate-severe CI, and 15% had depression. Moderate-severe CI (β=0.84, p< 0.01) and severe depression (β=1.31, p< 0.01) were each independently associated with IADL impairments in the adjusted model. The interaction term of moderate-severe CI with depression was also significant (β -0.85, p < 0.01). Depression significantly worsens the association of CI with IADL impairment, highlighting the importance of identifying and treating this common condition in older adults.

